# INTERACTION BETWEEN NATIVITY AND NEIGHBORHOOD ON DEMENTIA RISK: FINDINGS FROM TEN YEARS OF NATIONAL US DATA

**DOI:** 10.1093/geroni/igad104.0013

**Published:** 2023-12-21

**Authors:** Roger Wong, Daniel Soong

**Affiliations:** SUNY Upstate Medical University, Syracuse, New York, United States; SUNY Upstate Medical University, Syracuse, New York, United States

## Abstract

Prior research indicates neighborhood disadvantage increases dementia risk. There is, however, inconclusive evidence on the relationship between nativity and cognitive impairment. To our knowledge, our study is the first to analyze how nativity and neighborhood interact to influence dementia risk. Ten years of prospective cohort data (2011-2020) were retrieved from the National Health and Aging Trends Study, a nationally representative sample of 5,362 U.S. older adults aged 65+. Cox regression analyzed time to dementia diagnosis using nativity status (foreign or native-born) and composite scores for neighborhood physical disorder (litter, graffiti, vacancies) and social cohesion (know, help, trust each other), after applying sampling weights and imputing missing data. Average baseline neighborhood physical disorder was significantly higher among foreign-born (0.28) compared to native-born (0.18) older adults [t=-2.4, p=.02]. Average baseline neighborhood social cohesion was significantly lower for foreign-born (3.57) compared to native-born (4.33) older adults [t=5.5, p<.001]. After adjusting for sociodemographic, health, and neighborhood variables, foreign-born older adults had a 51% significantly higher dementia risk (aHR=1.51, 95% CI=1.19-1.90, p<.01). There were no statistically significant interactions for nativity with neighborhood physical disorder or social cohesion. Our findings suggest foreign-born older adults have higher neighborhood physical disorder and lower social cohesion compared to native-born older adults. Despite the higher dementia risk we observed for foreign-born older adults, this relationship was not moderated by either neighborhood physical disorder or social cohesion. Further research is needed to understand what factors are contributing to elevated dementia risk among foreign-born older adults.

